# Development of a blood sample detector for multi-tracer positron emission tomography using gamma spectroscopy

**DOI:** 10.1186/s40658-019-0263-x

**Published:** 2019-12-16

**Authors:** Carlos Velasco, Adriana Mota-Cobián, Jesús Mateo, Samuel España

**Affiliations:** 1 Centro Nacional de Investigaciones Cardiovasculares (CNIC), Madrid, Spain; 2 Universidad Complutense de Madrid; IdISSC, Madrid, Spain

**Keywords:** Positron emission tomography, Arterial input function, Multi-tracer PET, Gamma spectroscopy, Blood sample detector, Blood activity, Silicon photomultiplier, Pharmacokinetic modeling, Non-pure beta emitter

## Abstract

**Background:**

Multi-tracer positron emission tomography (PET) imaging can be accomplished by applying multi-tracer compartment modeling. Recently, a method has been proposed in which the arterial input functions (AIFs) of the multi-tracer PET scan are explicitly derived. For that purpose, a gamma spectroscopic analysis is performed on blood samples manually withdrawn from the patient when at least one of the co-injected tracers is based on a non-pure positron emitter. Alternatively, these blood samples required for the spectroscopic analysis may be obtained and analyzed on site by an automated detection device, thus minimizing analysis time and radiation exposure of the operating personnel. In this work, a new automated blood sample detector based on silicon photomultipliers (SiPMs) for single- and multi-tracer PET imaging is presented, characterized, and tested in vitro and in vivo.

**Results:**

The detector presented in this work stores and analyzes on-the-fly single and coincidence detected events. A sensitivity of 22.6 cps/(kBq/mL) and 1.7 cps/(kBq/mL) was obtained for single and coincidence events respectively. An energy resolution of 35% full-width-half-maximum (FWHM) at 511 keV and a minimum detectable activity of 0.30 ± 0.08 kBq/mL in single mode were obtained. The in vivo AIFs obtained with the detector show an excellent Pearson’s correlation (*r* = 0.996, *p* < 0.0001) with the ones obtained from well counter analysis of discrete blood samples. Moreover, in vitro experiments demonstrate the capability of the detector to apply the gamma spectroscopic analysis on a mixture of ^68^Ga and ^18^F and separate the individual signal emitted from each one.

**Conclusions:**

Characterization and in vivo evaluation under realistic experimental conditions showed that the detector proposed in this work offers excellent sensibility and stability. The device also showed to successfully separate individual signals emitted from a mixture of radioisotopes. Therefore, the blood sample detector presented in this study allows fully automatic AIFs measurements during single- and multi-tracer PET studies.

## Background

Positron emission tomography (PET) is a diagnostic molecular imaging technique that allows in vivo visualization of metabolic processes within the body based on the biodistribution of a radiotracer that is administered to the patient. Many applications of this technique are based on dynamic PET scans that provide access to the tracer kinetics in vivo. Furthermore, in many clinical cases, diagnostic accuracy can be increased considerably if complementary information is obtained from different tracers. For instance, in coronary artery disease (CAD) assessment, evaluation of both myocardial blood flow (MBF) and viability is usually required to provide an accurate diagnosis of the disease [1]. In these cases, it would be technically and economically advantageous to reduce the number of scans to the minimum. Several strategies have been proposed so far in order to enable the possibility of performing PET scans with multiple tracers simultaneously [2–6].

However, multi-tracer PET imaging may require obtaining the arterial input function (AIF) corresponding to each radiotracer involved in the scan. The main problem of explicit dual AIF determination is the undistinguishable nature of the annihilation photons coming from both radiotracers. Nonetheless, a spectroscopic analysis of the gamma emissions from blood samples containing two radiotracers could discriminate and separate both signals if at least one of the radiotracers contains a non-pure β^+^ emitter [7] that produces additional gamma emissions with an energy than can be distinguished from the annihilation photons. The feasibility of this technique has been previously demonstrated in cardiac PET studies with co-injection of ^68^Ga-DOTA and ^18^FDG for the determination of myocardial blood flow [8, 9], extracellular volume, and myocardial viability in a single scan [10].

This technique is based on gamma spectroscopic analysis of discrete blood samples manually withdrawn from the patient while multi-tracer PET scan is in progress. However, when obtained from manual blood sampling, AIFs are discrete and temporally limited by the rate at which the samples are obtained. Additionally, this method implies an increase of radiation exposure of the operating personnel and is more prone to errors as it requires rapid manual handling. To address these issues on single-tracer PET scans, several automatic blood sample detectors (hereinafter referred to as detectors) have been developed over the years that analyze on-the-fly the blood extracted from the patient during the PET acquisition. Existing detectors are based on the detection of either positrons [11, 12] or annihilation photons. Most of these gamma detection devices use scintillation crystals (such as BGO [13], GSO [14], NaI [15], or LYSO [16]) coupled to photomultiplier tubes (PMTs). However, alternatives have also been proposed, such as a CZT-based device [17] and LSO crystals coupled to an avalanche photodiodes (APDs) [18], which are more compact and can operate under magnetic fields.

The aim of this study was to develop a novel detector based on silicon photomultipliers (SiPMs) for AIF measurements on single- and multi-tracer PET imaging. The performance characteristics of the detector were evaluated and its capability for separation of dual AIFs using gamma spectroscopic analysis was tested. In addition, the accuracy of the detector and the capability to work under realistic experimental conditions were tested on in vivo studies.

## Methods

### Blood sample detector design

**Fig. 1:**
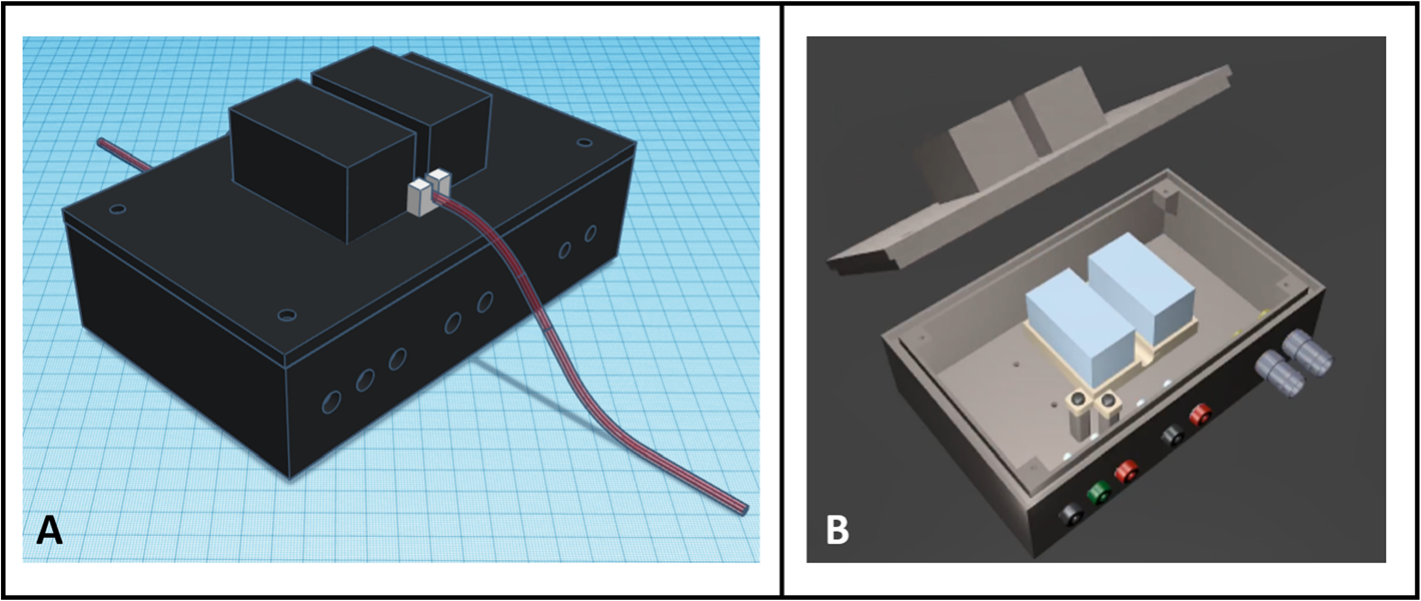
External (**a**) and internal (**b**) views of the blood sample detector developed in this study. The device consists of two 50 × 50 × 25 mm^3^ CsI(Tl) scintillation crystals (blue boxes) coupled on one of the 50 × 25 mm^2^ faces to a 4 × 4 mm^2^ SiPM (hidden in this illustration). Crystals are placed at 11.5-mm distance. The catheter (red tube) is placed in the gap between crystals with a holding cassette (white piece)

The proposed detector consists of two detection units, each one made with a 50 × 50 × 25 mm^3^ CsI(Tl) scintillation crystal with diffuse surfaces (Scionix Holland, Bunnik, The Netherlands) wrapped with a white diffuse plastic reflector. On the center of one of the 50 × 25 mm^2^ faces, an area of 4 × 4 mm^2^ is left unwrapped for optical readout. CsI(Tl) was selected for its high detection efficiency, high light yield, and absence of intrinsic radioactivity, which allows performing measurements in single and coincidence modes making it suitable for PET and SPECT radiotracers. Each crystal is coupled with optical grease to a SiPM (ASD-RGB4S-P, AdvanSiD, Trento, Italy) with an active area of 4 × 4 mm^2^ operated at 31 V. SiPMs are compact, MR compatible, can be operated at low voltage, and have a very high gain (10^6^). SiPMs are connected to amplification boards based on inverting transimpedance amplifiers (ASD-EP-EB-N - SiPM Evaluation Board, AdvanSiD, Trento, Italy). Crystals are placed side-by-side along one of the 50 × 50 mm^2^ faces with an 11.5-mm gap between them (see Fig. 1a). The crystals, SiPMs, and readout electronics are placed within a 3D-printed enclosure (see Fig. 1b). The blood sampling catheter is placed in the center of the gap between crystals in a 3D-printed U-grooved holding cassette. This piece can be fixed to the detector enclosure, allowing for measurements in single and coincidence modes and ensuring a good reproducibility. The detector shielding is made of a double layer of 3-cm-thick lead bricks in order to minimize the detection of external radiation.

### Data acquisition and signal processing

The signals from both detection units are digitized (15 MS/s) with an oscilloscope (Picoscope Series 2206A, Pico Technology Ltd, Cambridgeshire, UK) and sent to a PC for further processing using a custom-made application based on the oscilloscope’s C++ API. All pulses triggered by a falling edge discriminator with a threshold of −50 mV are integrated for 2 μs (no filters were applied) to obtain the energy deposited by the gamma photons and stored as single events for each detector. The signal is also recorded for 1 μs pre-trigger, and the average of those pre-trigger samples is used for baseline correction. Time stamps are generated by the leading edge discrimination in both detectors. Single events with a time difference below 300 ns are also stored as coincidence events. Random coincidences are estimated based on the single rates [19]. Environmental background events are subtracted from single and coincidence events. For that purpose, a 2-h measurement with no activity in the catheter was recorded.

### Device characterization

#### Sensitivity, energy resolution, coincidence resolving time and count rate losses

In order to characterize the detector, an 0.8-mm internal diameter (ID) catheter (Tygon S3 E-3603, Saint-Gobain Performance Plastics Co., Akron, OH) was filled with ^18^FDG at an initial activity concentration of 900 kBq/mL and placed in the detector with the active length centered with the detection units. A 15-h acquisition was performed consisting of 150 frames of 1-min duration with 5-min gap intervals. The energy spectra were obtained and calibrated using the 511-keV peak for every acquisition in order to compensate for temperature-dependent gain variations. The energy resolution at the 511-keV photopeak was obtained by fitting to a Gaussian.

Sensitivity was computed as the slope of the linear fit of the events rate (singles or coincidences) measured within an energy window of 350–700 keV against their corresponding activity concentration. This fit was performed for the low count rate measurements. Count rate losses were estimated for single events assuming that no events are lost at low count rate. Coincidence resolving time (CRT) was derived from the time difference histogram for coincidence events within the same energy window of 350–700 keV by interpolation between the two bins on each side of the maximum that are immediately above and below the half maximum.

#### Minimum detectable activity

Minimum detectable activity (MDA) determines the smallest activity concentration that can be detected with a certain confidence level [20]. MDA depends on sensitivity of the detector (*s*), the duration of the measurement (*T*), and background events (*N*_*B*_) detected during *T*. In terms of kBq/mL, and at a 95% confidence level, MDA can be described as:

1 $$ \mathrm{MDA}=\frac{4.65\ \sqrt{N_B+2.71}}{fTs} $$

where *f* is the branching ratio for β^+^ decays (0.967 for ^18^F). *N*_*B*_ was measured without and with the presence of an external source of activity. In the first case, only the environmental radiation contributes to background radiation, whereas in the second case, the radiation emitted from the patient itself can penetrate the shielding and contribute to background signal. Consequently, MDA was determined in both scenarios for both single and coincidence events using an energy window of 350–700 keV. Details about the setup employed for the background measurements with an external activity source are given in the following section. In each case, background measurements of 2–3 min were performed in three independent acquisitions. The results are presented as mean ± SD.

### In vivo evaluation

The performance of the detector was evaluated in vivo in animals injected with ^18^FDG. Three healthy female large white pigs (mean weight = 45 ± 4 kg) were anesthetized by intramuscular injection of ketamine (20 mg/kg), xylazine (2 mg/kg), and midazolam (0.5 mg/kg), and maintained by continuous intravenous infusion of ketamine (2 mg/kg/h), xylazine (0.2 mg/kg/h), and midazolam (0.2 mg/kg/h). Oxygen saturation levels via pulse oximetry, and electrocardiogram signal, were monitored throughout the study. The coccygeal artery of the animal was cannulated, and arterial blood was withdrawn through an 0.8-mm ID catheter at 5 mL/min using a peristaltic pump. The animals received a bolus injection of heparin and the catheter was washed with heparinized saline to prevent clotting. The animal was placed in a PET/CT Gemini TF-64 scanner (Philips Healthcare, Best, The Netherlands) and the scanner table was moved to the position were cardiac PET acquisitions are routinely performed. The detector was placed at about 40 cm from the animal’s tail in order to minimize blood dispersion inside tubing. ^18^FDG (155 ± 12 MBq) was prepared in 6 mL and infused at a rate of 1.0 mL/s through a marginal ear vein, followed by a 6-mL saline flush at the same rate. The acquisition with the detector started with the radiotracer injection and lasted for 5 min. The AIF was obtained with the detector (AIF_D_) in consecutive 5-s frames using the single events recorded in the 350–700 keV energy window and converted to activity concentration using the sensitivity previously obtained. Decay correction was applied. Afterwards, measurements for MDA determination with an external source of activity were performed by placing an empty catheter in the device and leaving everything else in the same position.

In order to validate AIF_D_ results, blood samples were collected into sample tubes after passing through the detector following this temporal scheme: 20 × 5 s, 8 × 10 s, 6 × 20 s. Then, the tubes were briefly centrifuged to provide a reproducible geometrical distribution of the blood and later analyzed using a well counter (Wallac 1470 Perkin Elmer, Waltham, Massachusetts, USA) applying dead time and decay corrections. The well counter was previously calibrated to convert measurements to activity values. The volume for each blood sample was determined as the weight difference between empty and filled tubes and applying a blood density of 1.03 g/mL [21]. Finally, the activity concentration of ^18^FDG was calculated for each blood sample obtaining the AIF derived from the well counter (AIF_WC_). The time delay existing between AIF_D_ and AIF_WC_ was corrected by maximizing the cross-correlation between both curves, which had been previously interpolated every 5 s.

### Multi-tracer AIF detection by spectroscopic analysis

**Fig. 2:**
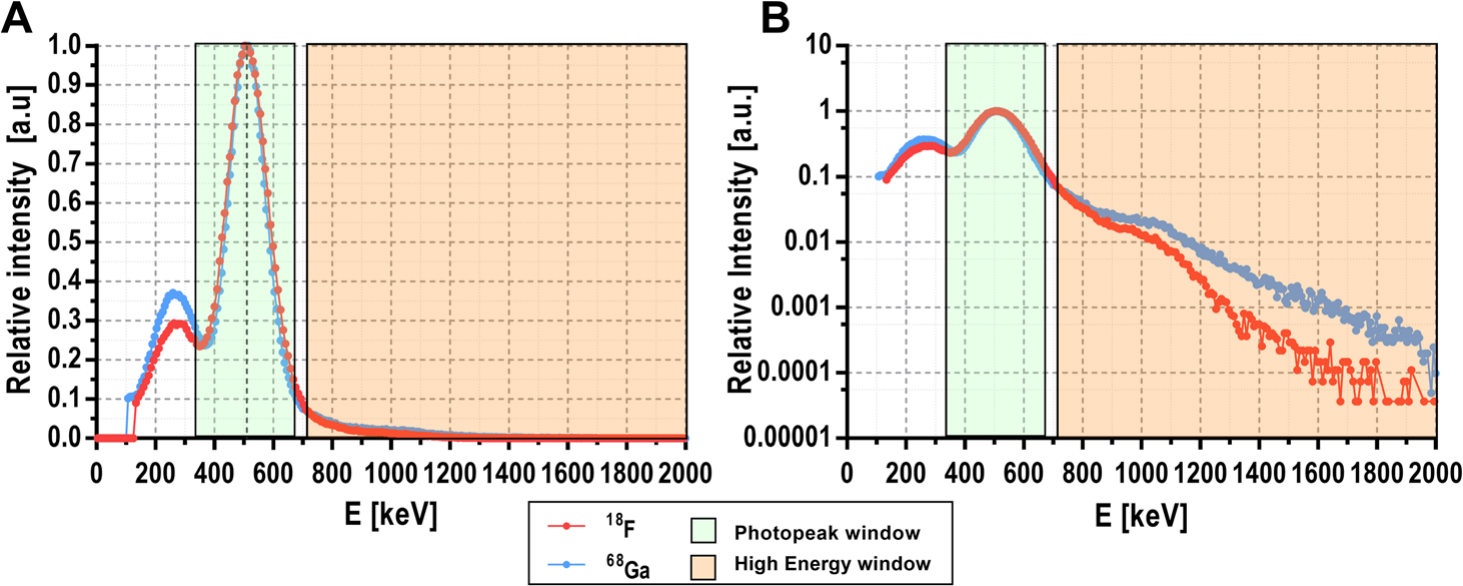
Normalized energy spectra of single events recorded from ^18^F (pure β^+^ emitter, red) and the ^68^Ga (non-pure β^+^ emitter, blue) shown in linear (**a**) and logarithmic (**b**) scales. The green and orange boxes represent the energy windows set at (350, 700) and (750, 2000) keV to distinguish between photopeak (low energy) and high-energy events. The events recorded in the high-energy window are relatively higher for ^68^Ga than for ^18^F compared with the event recorded in the photopeak

Pure β^+^ isotopes such as ^18^F only emit positrons that result in 511-keV annihilation photons, while non-pure β^+^ isotopes like ^68^Ga emit additional non-annihilation photons, although only those with 1.077 MeV are emitted in a significant fraction of the decays (3.22%) [22]. Thus, the ratio of events recorded at high-energy (> 750 keV) and low-energy windows (350–700 keV) (see Fig. 2) can be used to determine the amount of ^18^F and ^68^Ga in a sample containing any combination of both isotopes.

Two acquisitions were made in order to obtain the calibration data required to implement the proposed method. An 0.8-mm ID catheter was filled with ^68^Ga or ^18^F respectively with an initial activity concentration of 1200 kBq/mL (measured with an activimeter). Afterwards, the catheter was placed in the device and data was recorded for several hours acquiring frames of 1 min with 5-min gap intervals. Each frame was processed to obtain the total single rate at high-energy window (750–2000 keV, *S*_HE_) and the coincidences rate at low-energy window (350–700 keV, *C*_LE_). The ratio between *S*_HE_ and *C*_LE_ could be used, in theory, to calibrate the measurements for each isotope. However, we observed that, at higher count rates, the increase of *S*_HE_ is not linear with *C*_LE_ due to pile-up events. Therefore, in order to include this effect, *S*_HE_ must be calibrated as a function of *C*_LE_. For that purpose, the variation of *S*_HE_ against *C*_LE_ was represented and fitted to a third-degree polynomial for each isotope (*S*_HE,F_(*C*_LE_) and *S*_HE,G_(*C*_LE_) for pure ^18^F and ^68^Ga respectively). When measuring *S*_HE_ and *C*_LE_ for a sample containing an unknown mixture of ^18^F and ^68^Ga (*S*_HE,Mix_), the relative activity of each isotope (*a*_Ga,D_ and *a*_F,D_ where *a*_F,D *+*_ *a*_Ga,D_ = 1) can be obtained using the following expression:

2 $$ {S}_{\mathrm{HE},\mathrm{Mix}}\left({C}_{\mathrm{LE}}\right)={a}_{\mathrm{Ga},\mathrm{D}}\times {S}_{\mathrm{HE},\mathrm{G}}\left({C}_{\mathrm{LE}}\right)+\left(1-{a}_{\mathrm{Ga},\mathrm{D}}\ \right)\times {S}_{\mathrm{HE},\mathrm{F}}\left({C}_{\mathrm{LE}}\right) $$

assuming that the contribution of pile-up events is equally distributed between both isotopes.

The ability of the developed detector to obtain separated AIFs of tracers labeled with different isotopes (^68^Ga and ^18^F) was tested in vitro. To do so, a catheter filled with a mixture of 73% ^68^Ga and 27% ^18^F with an initial total activity concentration of 1200 kBq/mL was placed in the detector. The activity was measured separately for each isotope with an activimeter and the mixture was prepared afterwards. Data was recorded for several hours acquiring frames of 1 min with 5-min gap intervals. The relative activity of both isotopes was constantly changing over time due to their different half-lives (109 min for ^18^F and 68 min for ^68^Ga).

The initial activity concentration (*c*(*t*_i_) = 1200 kBq/mL) was much higher than the typical values that can be measured in an AIF of an in vivo experiment on large animals. In the later studies, the activity concentration usually ranges between 10 and 200 kBq/mL, and the duration of the time frames of the dynamic PET acquisition is frequently set from 5 s (in those frames where the activity concentration is high and changes rapidly) up to 60–180 s (for those frames where the activity concentration is low and the changes over time are less significative). Hence, in order to evaluate our experiment in more realistic conditions, our 1-min acquisitions were trimmed depending on the activity concentration of each acquisition as follows:

3 $$ \left\{\begin{array}{c}\kern1em t=60\ \mathrm{s}\kern2.25em ,\kern0.5em \mathrm{when}\kern4.75em \max\ \left(c(t)\right)\le 10\ \mathrm{kBq}/\mathrm{mL}\kern8.25em \\ {}\kern1em t=10\ \mathrm{s}\kern2.25em ,\kern0.5em \mathrm{when}\kern4.5em 10\ \mathrm{kBq}/\mathrm{mL}<\max\ \left(c(t)\right)\le 50\ \mathrm{kBq}/\mathrm{mL}\ \kern1.5em \\ {}\kern0.75em t=5\ \mathrm{s}\kern3em ,\kern0.5em \mathrm{when}\kern4.5em 50\ \mathrm{kBq}/\mathrm{mL}<\max\ \left(c(t)\right)\kern8em \end{array}\right. $$

*S*_HE,Mix_ and *C*_LE_ were obtained for each frame and *a*_Ga,D_ was calculated using Eq. (2). These results were compared against theoretical values (*a*_Ga,A_) obtained from their initial activity and taking into account their half-lives. The mean relative difference between *a*_Ga,D_ and *a*_Ga,A_ was obtained and reported as percentage.

## Results

### Detector performance and in vivo evaluation

**Fig. 3:**
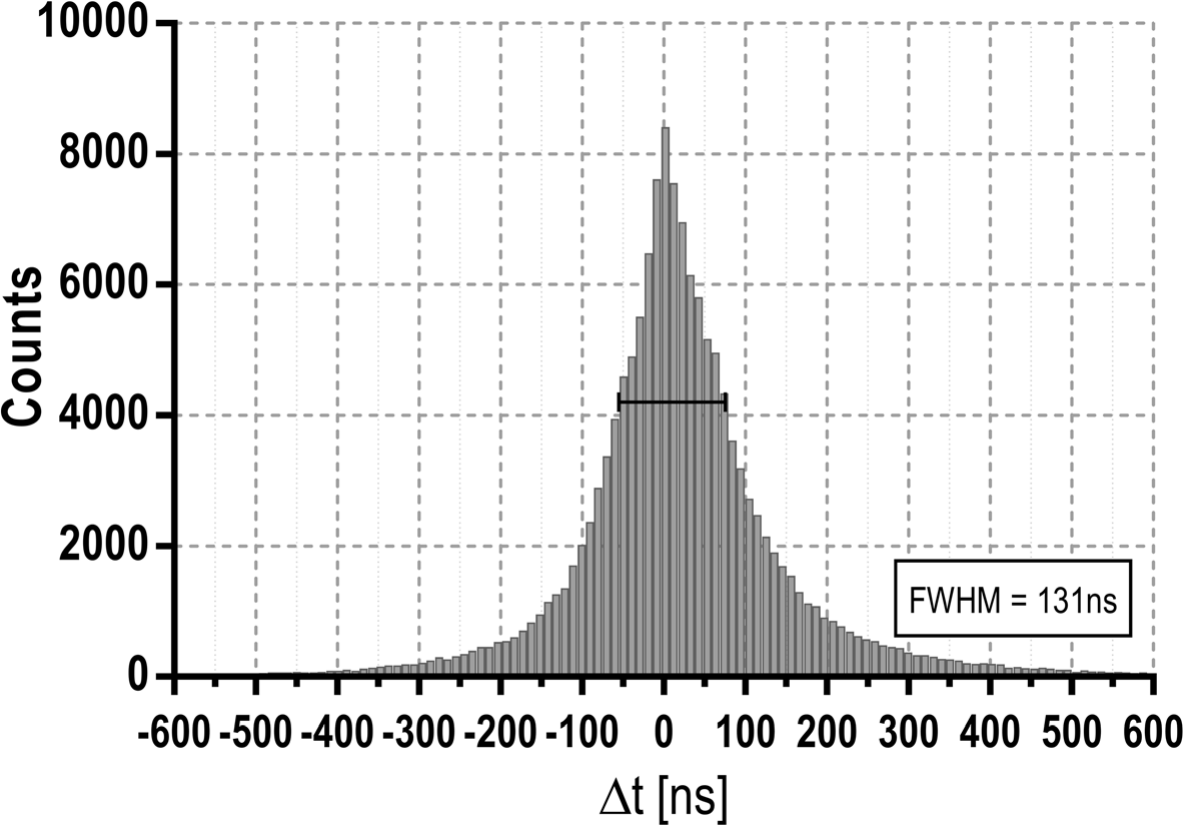
Time difference histogram obtained with the detector using ^18^F for a 1-min acquisition with an energy window of 350–700 keV leading to a CRT of 131 ns FWHM

**Fig. 4:**
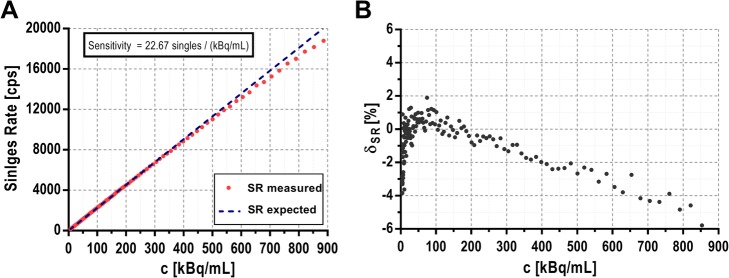
**a** Single rate (SR) detected by the detector as a function of activity concentration (c, red dots) inside the catheter. A linear fit of the measured SR at low c when count rate losses are negligible is also shown (blue dashed line). **b** Count rate losses (δSR) as a function of activity concentration

The energy resolution and the CRT of the device was determined using one of the acquisitions performed at low count rate, obtaining 35% FWHM at 511 keV (see Fig. 2) and 131 ns FHWM (see Fig. 3) respectively. The count rate losses for single events are shown in Fig. 4. Figure 4a displays the recorded single rate as a function of the activity concentration within the catheter and Fig. 4b illustrates the deviation of measured single rate from the expected value as a function of the activity concentration. Importantly, for activity concentrations within the range of standard in vivo experiments (10–200 kBq/mL), the count rate losses are lower than 2%. Random coincidences were also computed and can be considered negligible (< 0.3%). The sensitivities obtained for single and coincidence events were 22.6 cps/(kBq/mL) and 1.7 cps/(kBq/mL) respectively.

The MDA was computed for single and coincidence events with and without an external activity source. In the absence of an external source, the MDA was 0.05 ± 0.02 (singles) and 0.10 ± 0.03 kBq/mL (coincidences), while the results with an external source were 0.30 ± 0.08 kBq/mL (singles) and 0.25 ± 0.05 kBq/mL (coincidences).

**Fig. 5:**
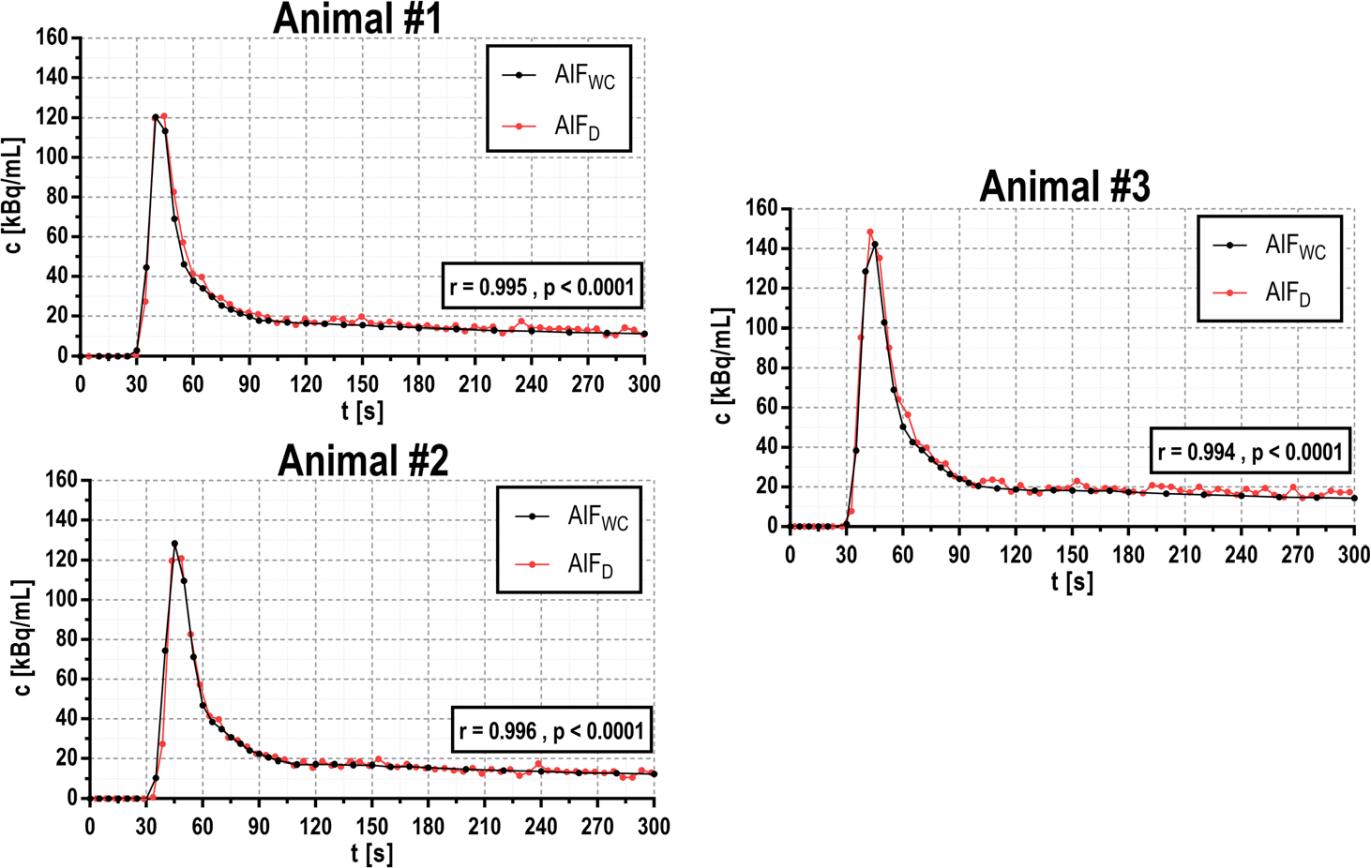
AIFs obtained with the detector (AIFD, red curves) compared with the AIFs obtained from collected blood sampling analyzed in a well counter (AIFWC, black curves) on three pigs that were injected with 18FDG. Pearson’s correlation obtained in all cases is excellent (mean *r* = 0.996, *p* < 0.0001).

Figure 5 shows the AIFs obtained with our detector (AIF_D_) from three in vivo studies in which blood was withdrawn at a rate of 5 mL/min from an 0.8-mm ID catheter. An excellent correlation between AIF_D_ and the blood samples analyzed with the well counter (AIF_WC_) was found (*r* = 0.996, *p* < 0.0001).

### Multi-tracer AIF separation by spectroscopic analysis

**Fig. 6:**
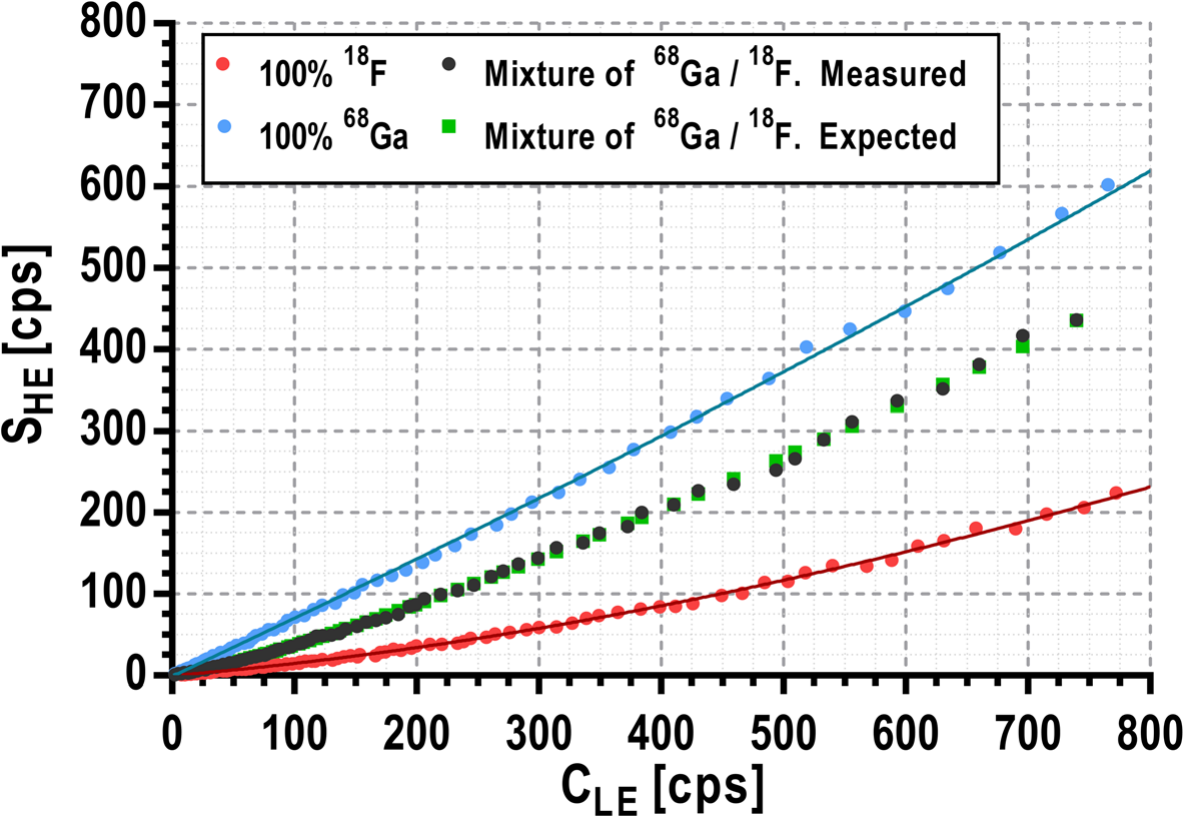
Single rates within the high-energy window (*S*_HE_) as a function of the coincidence rate within the low-energy window (*C*_LE_) for measurements performed with pure ^18^F (red dots), pure ^68^Ga (blue dots), and a mixture of both radioisotopes (black dots). The results for the pure samples were fitted to a third-degree polynomial (blue and red lines for pure ^68^Ga and ^18^F respectively). These fits are used as the calibration curves named as *S*_HE,F_ and *S*_HE,Ga_ in Eq. (2). Green squares represent the expected values for the mixture obtained using Eq. (2) and the theoretical relative activity (*a*_Ga,A_)

Figure 6 shows the calibration curves performed for the in vitro measurements of pure ^18^F and pure ^68^Ga (*S*_HE,F_ and *S*_HE,Ga_ respectively) as well as the high energy single rate corresponding to an unknown mixture of these radioisotopes (*S*_HE,Mix_). It can be observed that the *S*_HE,Mix_ dataset gets closer to the pure ^18^F measurements for lower *C*_LE_ since ^18^F activity decreases slower than ^68^Ga and consequently the relative activity of ^18^F increases over time. All datasets tend to *S*_HE_ = 0 at low activity concentrations, which confirms that the background subtraction to every acquisition has been correctly performed.

**Fig. 7:**
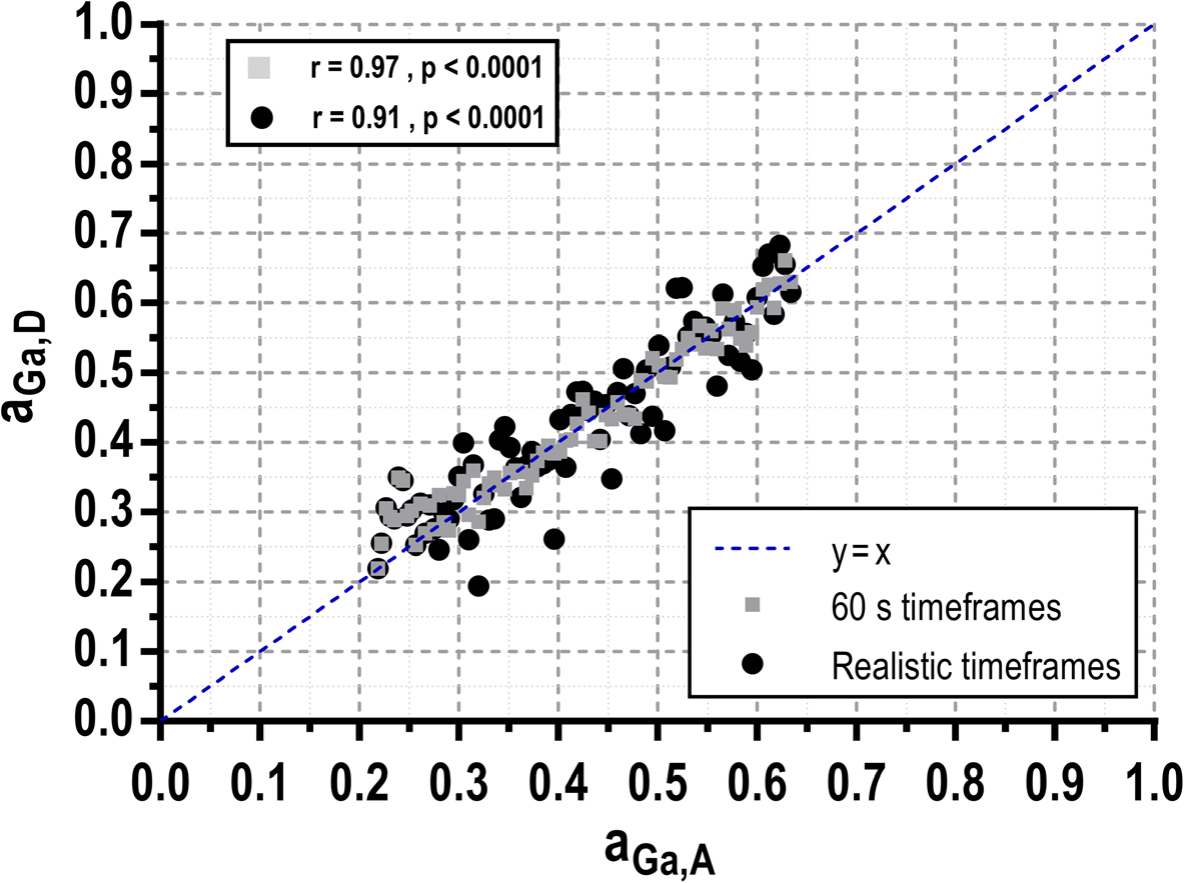
Comparison between the relative activity of 68Ga obtained with the detector using the gamma spectroscopy analysis of a mixture of ^68^Ga and ^18^F (aGa,D) and the theoretical relative activity (aGa,A). The data collected from the 1-min acquisitions were analyzed entirely (gray squares) and partially (following scheme given in Eq. (3), black dots). Pearson’s *r* correlation of the datasets with the true values (aGa,A) are *r* = 0.97 and *r* = 0.91 respectively. Dashed blue line represents the identity line

The relative activity of ^68^Ga in the mixture was calculated using Eq. (2) and the comparison against the theoretical values showed a very strong correlation (*r* = 0.97, *p* < 0.0001, Fig. 7). The mean relative difference between *a*_Ga,D_ and *a*_Ga,A_ was *δ* = (3 ± 10)%. When the acquisition was trimmed as described in Eq. (3) to analyze more realistic activity concentrations and time frame duration, the correlation was still very good (*r* = 0.91, *p* < 0.0001), with only a slight increase in the variation of the mean deviation from the true values (*δ* = (2 ± 13)%).

## Discussion

Our results show that the detector presented in this study has a high sensitivity and it can obtain accurate and stable measurements of AIFs under realistic conditions. The device can work within a wide range of activity concentrations (up to 400 kBq/mL with dead time losses below 2%, Fig. 4) and exhibits a very low MDA for both single and coincidence events (0.25 and 0.30 kBq/mL respectively) in the presence of an external activity source. Therefore, the detection limit of this device is sufficiently low to measure typical input functions observed in human or clinical cases, which typically range from 5 to 200 kBq/mL. Furthermore, the device is potentially MR compatible thanks to the SiPM-based detection system, although further work (such as mechanical and electronic components) would be required to enable this option.

The in vivo performance of the detector has been evaluated with three pigs leading to an excellent correlation between the AIFs obtained with the device and those obtained from the analysis of collected blood samples in a well counter (mean correlation *r* = 0.996, all with *p* < 0.0001).

The configuration parameters used in these studies, such as activity concentrations, tubing size, and time frame scheme are similar to those used in human studies. Therefore, this device would be suitable for application in human studies. In case of studies with rats, the inner diameter of the tubing usually ranges from 0.18 to 0.64 mm, which translates in a reduction of 60 to 95% of the active volume. However, the injected dose per gram for rats is about 30 times higher than for humans. Thus, our device could also be used in rat studies since similar count rate to that obtained in pig studies is expected. Moreover, the blood withdrawal rate could be reduced proportionally to the catheter volume reduction [18] while keeping a similar blood renewal rate in the active length of de device. For example, for a 0.18-mm ID tubing, blood could be withdrawn at a rate of 0.25 mL/min with similar accuracy to the results presented in this work.

When measuring AIF with a blood sampling detector, blood activity dispersion inside the tubing reveals as a critical factor that may introduce large errors in the final quantification. Many approaches have been proposed to correct for dispersion [23–25] analytically, but it is still advisable to minimize the main sources of dispersion in the experimental setup, such as tubing length or blood withdrawal rate. In this aspect, the compact design of our detector as a consequence of the use of SiPM detectors makes possible to place it in close proximity to the subject, which turns into a reduced blood dispersion effect. Furthermore, the capability of the detector to record coincidence events reduces the background events from external sources.

To our knowledge, available blood sample detectors can only measure single-tracer AIFs. In contrast, the detector presented in this work not only provides the live photon count rate emitted from the blood but also stores and analyses the gamma energy spectrum of each event recorded during the acquisition (both in single and coincidence modes). This feature allows obtaining individual tracer AIFs on multi-tracer PET studies by using the blood spectroscopic analysis described in [10].

The gamma spectroscopic analysis technique has been successfully implemented and validated in our detector for its application in multi-tracer PET studies when one of the radiotracers is based on a non-pure β^+^ emitter. The analysis for multi-tracer AIF detection was tested using the detector with mixtures of a pure β^+^ emitter (^18^F) and a non-pure β^+^ emitter (^68^Ga). The detection of additional gamma photons emitted by ^68^Ga (see Fig. 4) enabled to disentangle the activity of each radioisotope. The energy resolution (35% FWHM at 511 keV) and CRT (131 ns FHWM) obtained for our detector are poor compared with other detectors [16–18]. These results are due to the large difference between the size of the SiPMs (4 × 4 mm^2^) and the size of the scintillation crystal (50 × 50 × 25 mm^3^). Although the energy resolution is not a critical factor for standard blood sampling analysis, it may limit the accuracy for blood sampling spectroscopy. However, validation results shown in Fig. 7 demonstrate the capability of the developed device to separate the contribution of ^18^F and ^68^Ga for a sample containing a mixture of both isotopes with a very good correlation (*r* = 0.91, *p* < 0.0001). Nevertheless, the CRT did not affect the device performance as the obtained fraction of random events was very low in all cases.

## Conclusions

We have developed a blood sample detector based on SiPM technology capable of recording simultaneously single and coincidence events with different energy windows. The performance parameters of this detector have been obtained leading to excellent results in terms of sensitivity and MDA compared with other devices presented in the literature [17]. The detector was tested in vivo in pigs injected with a single radiotracer and the results were validated showing excellent results. Therefore, the device presented in this study allows performing fully automatic single- and multiple-tracer PET studies. Moreover, this is the first blood sample detector based on SiPM technology which offers many advantages in terms of cost, compactness, performance, and MR compatibility.

## Data Availability

Data and materials are available on request to the authors.

## Abbreviations

AIF: Arterial input function
AIF_D_: Arterial input function obtained with the detector
AIF_WC_: Arterial input function derived from analysis of blood samples with a well counter
CRT: Coincidence resolving time
CT: Computed tomography
DOTA: 1,4,7,10-Tetraazacyclododecane-1,4,7,10-tetraacetic acid
FDG: Fludeoxyglucose
FWHM: Full width at half maximum
ID: Internal diameter
MDA: Minimum detectable activity
PET: Positron emission tomography
SiPM: Silicon photomultiplier
